# Determinants of early initiation of breastfeeding in rural Tanzania

**DOI:** 10.1186/s13006-015-0052-7

**Published:** 2015-09-25

**Authors:** Amon Exavery, Almamy Malick Kanté, Ahmed Hingora, James F. Phillips

**Affiliations:** Ifakara Health Institute, Plot 463, Kiko Avenue, P.O. Box 78373, Mikocheni, Dar es Salaam Tanzania; Department of Population and Family Health, Mailman School of Public Health, Columbia University, New York, USA

**Keywords:** Breastfeeding, Timely initiation, Early initiation, Prevalence, Predictors, Rufiji, Kilobero, Ulanga, Tanzania

## Abstract

**Background:**

Breastfeeding is widely known for its imperative contribution in improving maternal and newborn health outcomes. However, evidence regarding timing of initiation of breastfeeding is limited in Tanzania. This study examines the extent of and factors associated with early initiation of breastfeeding in three rural districts of Tanzania.

**Methods:**

Data were collected in 2011 in a cross–sectional survey of random households in Rufiji, Kilombero and Ulanga districts of Tanzania. From the survey, 889 women who had given birth within 2 years preceding the survey were analyzed. Both descriptive and inferential statistical analyses were conducted. Associations between the outcome variable and each of the independent variables were tested using chi–square. Logistic regression was used for multivariate analysis.

**Results:**

Early initiation of breastfeeding (i.e. breastfeeding initiation within 1 h of birth) stood at 51 %. The odds of early initiation of breastfeeding was significantly 78 % lower following childbirth by caesarean section than vaginal birth (adjusted odds ratio (OR) = 0.22; 95 % confidence interval (CI) 0.14, 0.36). However, this was almost twice as high for women who gave birth in health facilities as for those who gave birth at home (OR = 1.75; 95 % CI 1.25, 2.45). Furthermore, maternal knowledge of newborn danger signs was negatively associated with early initiation of breastfeeding (moderate vs. high: OR = 1.73; 95 % CI 1.23, 2.42; low vs. high: OR = 2.06; 95 % CI 1.43, 2.96). The study found also that early initiation of breastfeeding was less likely in Rufiji compared to Kilombero (OR = 0.52; 95 % CI 0.31, 0.89), as well as among ever married than currently married women (OR = 0.46; 95 % CI 0.25, 0.87).

**Conclusions:**

To enhance early initiation of breastfeeding, using health facilities for childbirth must be emphasized and facilitated among women in rural Tanzania. Further, interventions to promote and enforce early initiation of breastfeeding should be devised especially for caesarean births. Women residing in rural locations and women who are not currently married should be specifically targeted with interventions aimed at enhancing early initiation of breastfeeding to ensure healthy outcomes for newborns.

## Background

To ensure the health of newborns, the World Health Organization (WHO) recommends placing newborn babies in direct contact with their mothers immediately after birth for at least 1 h, and encouraging initiation of breastfeeding as soon as possible [1]. Evidence shows that if newborns are exposed to breastfeeding at this early stage, they are much more likely to adhere to exclusive breastfeeding and ultimately have better health outcomes [2, 3]. Exclusive breastfeeding contributes to a 22 % reduction in neonatal mortality [4], suggesting that the earlier breastfeeding initiation takes place, the lower the chances of neonatal mortality, mostly due to reduction in infection [5]. A recent systematic review regarding timing of breastfeeding initiation shows that initiating breastfeeding within 1 h of birth results in significant reduction of the risk of neonatal mortality [6]. One ecological study that uses demographic and health survey (DHS) data from 67 countries shows a protective effect of breastfeeding within the first hour of birth on neonatal survival [7]. Several other studies indicate similar results regarding the importance of early initiation of breastfeeding [4, 8].

The benefits of early initiation of breastfeeding extend to both the mother and the newborn. Consistent production and ejection of breast milk is facilitated by secretion of prolactin and oxytocin which are stimulated by suction of the nipple by the newborn, thus indicating that early initiation of breastfeeding aids in consistent breastfeeding. The first breast milk to be secreted for the first few days after birth, known as colostrum, provides the newborn with natural immunity from the mother to avert infections. Therefore, it is particularly important to the health of newborns that they are breastfed immediately after birth [9]. Breast milk has immunological properties which protect newborn babies against common illnesses and infectious diseases such as diarrhea [10, 11], respiratory infections especially pneumonia [12], meningitis [10, 11] and neonatal sepsis [13–16], all of which are important causes of infant morbidity and mortality.

In addition to these benefits, early suckling stimulates the contraction of the uterus after childbirth which reduces risk of postpartum hemorrhage [9]. Furthermore, breastfeeding lengthens the postpartum infertility period, helps the mother return to her pregestational weight, and reduces the risk of breast cancer [17] as well as ovarian cancer [18–20].

While the benefits of early initiation of breastfeeding are well recognized, a significant proportion of newborn babies in many countries are still not breastfed within 1 h of birth as the WHO recommends. This implies that barriers still exist, thus necessitating further investigations. In Tanzania, the recent DHS report shows that less than a half (49 %) of newborn babies are breastfed within 1 h of birth, and ranges from 18 % in Rukwa region to 93 % in Manyara region [9]. However, 94 % of the Tanzanian newborns are breastfed within 1 day after birth [9]. In the Sub-Saharan African region, prevalence of early initiation of breastfeeding varies widely, with less than 60 % of mothers in Kenya [21], Ethiopia [22] and Lesotho [23] initiating breastfeeding within the first hour; and as many as 95 % of women in Malawi adhering to this recommendation [24]. Elsewhere, early initiation of breastfeeding has been estimated at 11 % in one province in Saudi Arabia [25], 31 % in India [26] and 83 % in Sri Lanka [27].

Several factors associated with early initiation of breastfeeding have been identified. This includes absence of prelacteal feeding, rural residence, absence of breast problems, and multi-parity [25]. One recent systematic review found that childbirth by caesarean section is the most consistent risk factor for delayed breastfeeding initiation [8]. Other factors identified by this review were low family income, maternal age under 25 years, low maternal education, absence of prenatal health visits, home birth, absence of prenatal guidance on breastfeeding, and preterm birth. In Sri Lanka, delayed initiation of breastfeeding was associated with low birth weight and childbirth by caesarean section, but less likely among female infants and mothers within higher wealth quintiles [27]. Furthermore, one study in India found that the practice is influenced by educational level, economic status, mother’s tribe, place of birth, prenatal visits to health care facilities, assistance during childbirth, and partner’s violent behavior [26]. Unfortunately, Tanzania-specific literature on timing of initial breastfeeding is scarce. One large cross-sectional survey was conducted in Southern Tanzania regarding newborn care practices that reported prevalence of breastfeeding initiation within 1 h, yet this study did not assess factors associated with the behavior [28]. Only the recent DHS report for Tanzania points out that early initiation of breastfeeding increases with maternal educational status, wealth quintile, urban residence, and professional or skilled birth attendance [9]. Given that the proportion of women who breastfeed their newborns within 1 h of birth varies widely across Tanzania [9], and the lack of district level data on this behavior, it is possible that factors underlying the behaviour differ by geographical locations due to differences in context specific conditions. Therefore the current analysis assesses the levels and factors associated with early initiation of breastfeeding among women who have recently given birth in Rufiji, Kilombero, and Ulanga districts of Tanzania.

## Methods

### Study area

Data for this study were collected in three rural and impoverished districts of Rufiji, Kilombero and Ulanga in Tanzania using health and demographic surveillance system (HDSS) platforms of Rufiji [29] and Ifakara [30]. The Rufiji HDSS is located in the Rufiji district of the Pwani region, while the Ifakara HDSS sits on geographical portions of both Kilombero and Ulanga districts in Morogoro region. A HDSS is a longitudinal, health and vital events registration system that monitors demographic events, such as births, deaths, and migrations in a geographically defined setting of individuals. During the survey in 2011, these HDSS were following up a combined population of about 370,000 people.

### Study design, sampling and study participants

This study is cross–sectional in design. It is based on a household survey which was carried out in 2011 in the study area to cater for baseline needs of the Connect Project [31, 32]. The project tests a child survival impact and health behavior change of adding paid community health workers known as community health agents (CHAs) to an existing facility focused health system. This is happening in Rufiji, Kilombero and Ulanga districts of Tanzania. A sampling frame of households was obtained from the HDSS and probability proportionate to size (PPS) technique was used to select the sample. This ensured sample representativeness because the villages varied remarkably in terms of their population sizes. During the survey, a total of 2183 households were successfully visited. These households produced 3127 women who were interviewed, out of which 889 were eligible, hence selected for the current analysis. More details about the survey are available elsewhere [32].

### Variables

This study assessed one binary outcome variable, namely, early initiation of breastfeeding. According to WHO, early initiation of breastfeeding means providing mother’s breast milk to infants within 1 h of birth [33]. The variable had two categories, “yes” and “no”, which were assigned codes of “1” and “0” respectively for computational purposes. This variable was derived from the original fielded question which stated “how long after birth did you first put (name of the child) to the breast?” Responses to this question were depicted in number of hours, except that if the child was breastfed within 1 h of birth it was recorded as zero. At last, the outcome variable took the following form:

$$ \mathrm{Early}\ \mathrm{initiation}\ \mathrm{of}\ \mathrm{breastfeeding} = \left\{\begin{array}{c}\hfill 1\ \mathrm{if}\ \mathrm{breastfeeding}\ \mathrm{was}\ \mathrm{initiated}\ \mathrm{within}\ \mathrm{on}\mathrm{e}\ \mathrm{hour}\ \mathrm{of}\ \mathrm{birth}\ \hfill \\ {}\hfill 0\ \mathrm{if}\ \mathrm{breastfeeding}\ \mathrm{began}\ \mathrm{on}\ \mathrm{or}\ \mathrm{after}\ \mathrm{the}\ \mathrm{first}\ \mathrm{hour}\ \mathrm{of}\ \mathrm{birth}\ \hfill \end{array}\right. $$

Several independent variables were assessed. This included maternal age (in years), marital status, education attained, ethnic group, religion, household socioeconomic status which was constructed using principal component analysis (PCA) of household assets [34], district of residence, type of residence, gravidity, and pregnancy wantedness. Others were mode of childbirth, place of birth, number of times a woman received antenatal care (ANC) during pregnancy, whether or not during pregnancy a woman was counseled on immediate breastfeeding after birth, mother’s knowledge of health practices related to pregnancy, and mother’s knowledge of newborn danger signs.

The variable depicting mother’s knowledge of health practices related to pregnancy was constructed from seven questions which asked whether or not during pregnancy a woman was counseled on: (a) financial preparation for childbirth (b) Breastfeeding immediately after birth (c) Danger signs during childbirth (d) Using a skilled birth attendant (e) Family planning (f) Identifying emergency transport options and (g) Danger signs of pregnancy. A “yes” response to each of these questions was given a score of “1”, and a score of “0” if the response was “no”. Total number of the services for which the mother received counseling about was calculated for each woman, with possible values ranging from zero if none of the services was received to seven if all services were received. Finally three categories were constructed from the values, such that all women who scored seven were grouped together and defined as of comprehensive knowledge of health practices related to pregnancy. Women who scored between three and six inclusive were considered as having moderate knowledge, and those who had less than three scores were considered as having low knowledge of health practices related to pregnancy. This approach was similarly applied in the construction of the mother’s knowledge of newborn danger signs variable. Each woman was asked to mention any danger signs or symptoms which show that after birth a newborn baby needs health care. Reported danger signs were: (a) Fever (b) Unable to suckle/feed (c) Difficult/fast breathing (d) Diarrhea (e) Convulsions (f) Persistent vomiting (g) Yellow palms/soles/eyes/jaundice (h) Lethargy (i) Red/discharging eyes (j) Skin pustules (k) Skin around cord red (l) Pus from cord (m) Failure to pass urine (n) Shivering/cold baby/low temperature (o) Bluish palms and soles (p) Very small baby/below normal weight and (q) Others. Women who reported at least three of these danger signs were defined as having high knowledge of newborn danger signs. Women with moderate knowledge of newborn danger signs referred to those who reported only two danger signs, and those who reported less than two danger signs were defined as having low knowledge.

### Statistical analysis

Data analysis was both descriptive and inferential, and was carried out using STATA statistical software (version 11). Frequencies were calculated in one-way tabulations to reveal distributional features of the data across each variable. Bivariate analysis was then conducted by cross-tabulating early initiation of breastfeeding against each of the independent variables. In this process, the degree of association between each pair of cross-tabulated variables was tested using chi-square test (*χ*2) because all variables involved in the analysis were categorical. Logistic regression was used to perform multivariate analysis (regression model with more than one independent variable) in order to identify independent factors associated with early initiation of breastfeeding. Using log–likelihood ratio test, a variable was selected for inclusion in the multivariate model if it was statistically significant at 5 % level that its presence improved the overall model [35]. The model was also checked for statistical interactions and adequacy. Adequacy was checked using the Hosmer–Lemeshow goodness–of–fit test [35]. Adjusted odds ratios (OR) and their corresponding 95 % confidence intervals (CIs) and *p*-values were presented.

### Ethical consideration

A survey which produced these data received an ethical approval from both the Medical Research Coordinating Committee (MRCC) of the National Institute for Medical Research (NIMR) (NIMR/HQ/R.8a/Vol.IX/1203) and the Ifakara Health Institute’s Institutional Review Board (IRB) (IHI/IRB/No. 16–2010) in Tanzania. Ethical approval was also granted by the IRB of Columbia University, USA (Protocol AAAF3452). All respondents participated in the survey voluntarily, and signed an informed consent form prior to being interviewed. Completed questionnaires and consent forms were stored separately to ensure anonymity of the data.

## Results

### Profile of respondents

As Table 1 shows, the 889 women who were analyzed had an average age of 27.8 (±7.6) years. A majority of them (79.9 %) were married. In terms of education, 25 % had never been to school, 70.3 % had primary education, and only 4.7 % had secondary education or higher. Kilombero district represented more than half (58.5 %) of the respondents, while Rufiji and Ulanga had 24.9 and 16.7 % of the respondents respectively. The majority (81.1 %) of the respondents resided in rural settings. Of all these women, 12.9 % gave birth by caesarean section and the rest vaginally.

**Table 1:** Background characteristics of respondents analyzed for timing of breastfeeding initiation in rural Tanzania (*n* = 889)

	Absolute number of respondents (n)	Percent (%)
Overall	889	100.0
Maternal age (years)		
< 20	132	14.9
20–34	548	61.6
>34	209	23.5
Mean = 27.8, Min = 15, Max = 51	−	−
Marital status		
Married	710	79.9
Ever married	49	5.5
Single	130	14.6
Education attained		
None	222	25.0
Primary	625	70.3
Secondary+	42	4.7
Ethnic group		
Ndengereko	137	15.4
Ngindo	126	14.2
Pogoro	127	14.3
Sukuma	102	11.5
Other	397	44.7
Religion		
Muslim	452	50.8
Christian	379	42.6
Traditional/other	58	6.5
Socioeconomic status		
Poor	405	45.6
Middle	284	32.0
Rich	200	22.5
District of residence		
Kilombero	520	58.5
Rufiji	221	24.9
Ulanga	148	16.7
Type of residence		
Township/suburban	168	18.9
Rural	721	81.1
Pregnancy wantedness		
Intended	456	51.3
Mistimed	299	33.6
Unwanted	134	15.1
Mode of childbirth		
Vaginal	774	87.1
Caesarean section	115	12.9
Place of childbirth		
Home	227	25.5
Health facility	662	74.5

### Early initiation of breastfeeding by background characteristics

**Fig. 1:**
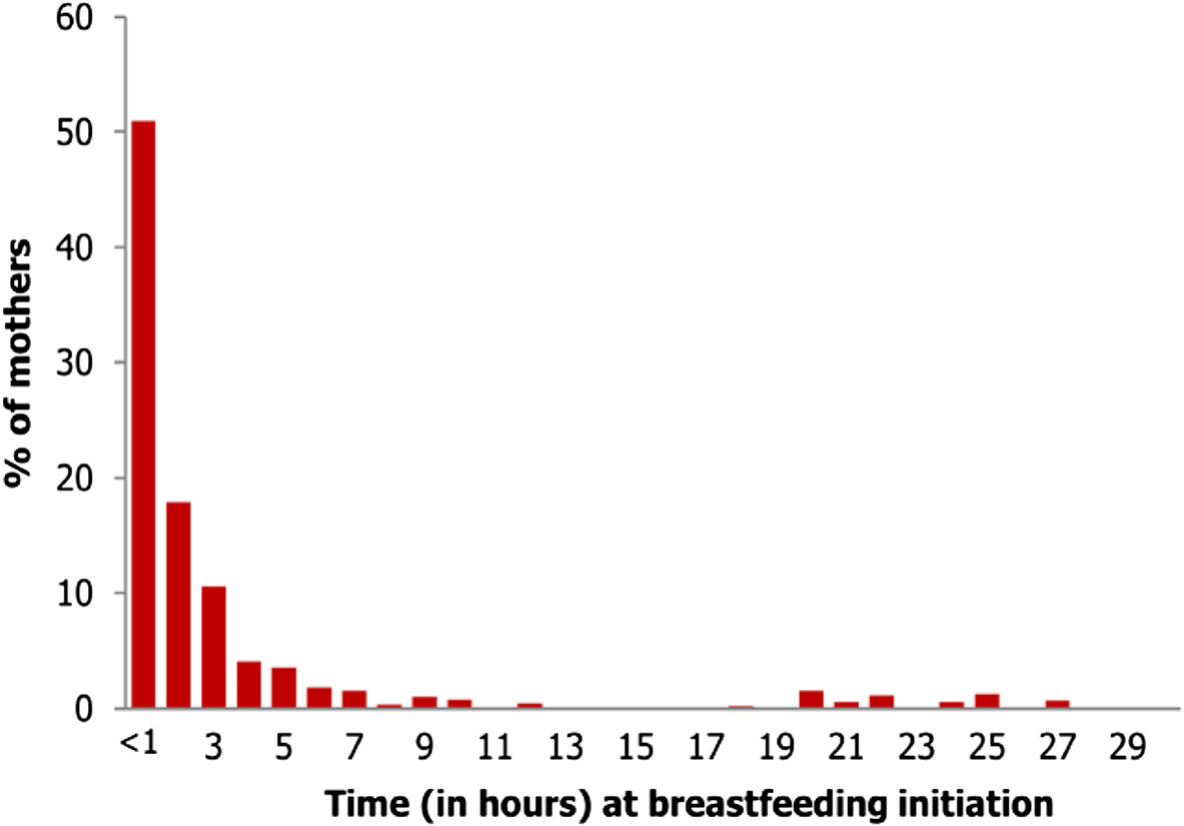
Percentage distribution of 889 women who have recently given birth in three rural districts of Tanzania according to the time elapsed after childbirth at which they initiated breastfeeding of their newborns babies

Overall, 51 % of the respondents initiated breastfeeding of their newborn babies within 1 h of birth as recommended by the WHO. By the end of the first day following childbirth, 94 % of the women had already initiated breastfeeding (Fig. 1). The proportion of women initiating breastfeeding within 1 h varied significantly by some characteristics of the respondents. The most important factor was mode of childbirth, whereby early initiation of breastfeeding became 54.4 % among women who gave birth vaginally, and 27.8 % among women who gave birth by caesarean section (*p* < 0.001). Also early initiation of breastfeeding was significantly higher among women who gave birth in health facilities than those who gave birth at home (53.9 % against 42.3 %). Mother’s knowledge of newborn danger signs was a significant variable in an opposite direction, such that early initiation of breastfeeding was lowest at 43.1 % among women with high knowledge and highest at 57.5 % among women with low knowledge of newborn danger signs.

Furthermore, ethnicity was also associated with early initiation of breastfeeding, whereby the lowest proportion was observed among Sukuma women (31.4 %) and the highest among Pogoro women (59.1 %). Similarly, religion was also significant, with early initiation of breastfeeding being 53.5 % among Muslim women, 51.7 % among Christian women and 29.3 % among women with traditional or other unspecified religions (*p* = 0.002). Other variables showed no significant association with early initiation of breastfeeding (Table 2).

**Table 2:** Breastfeeding initiation within 1 h of birth by each independent variable (*n* = 889)

	Absolute number of respondents (n)	% of women breastfed their newborns within 1 h of birth	*p*-value
OVERALL	889	51.0	−
Maternal age (years)			
<20	132	54.6	0.494
20–34	548	49.5	
>34+	209	52.6	
Marital status			
Married	710	51.6	0.212
Ever married	49	38.8	
Single	130	52.3	
Education attained			
None	222	46.9	0.162
Primary	625	51.7	
Secondary+	42	61.9	
Ethnic group			
Ndengereko	137	51.1	<0.001
Ngindo	126	50.0	
Pogoro	127	59.1	
Sukuma	102	31.4	
Other	397	53.7	
Religion			
Muslim	452	53.5	0.002
Christian	379	51.2	
Traditional/other	58	29.3	
Socioeconomic status			
Poor	405	48.6	0.283
Middle	284	51.1	
Rich	200	55.5	
District of residence			
Kilombero	520	53.9	0.095
Rufiji	221	48.4	
Ulanga	148	44.6	
Type of residence			
Township/suburban	168	57.1	0.075
Rural	721	49.5	
Pregnancy wantedness			
Intended	456	49.8	0.087
Mistimed	299	48.8	
Unwanted	134	59.7	
Mode of childbirth			
Vaginal	774	54.4	<0.001
Caesarean section	115	27.8	
Place of childbirth			
Home	227	42.3	0.002
Health facility	662	53.9	
Gravidity			
1	181	55.8	0.325
2–4	422	50.2	
>4	286	49.0	
Number of ANC visits made			
<4	507	52.1	0.444
≥4	382	49.5	
Counseled on immediate breastfeeding during ANC visits?			
No	276	48.9	0.414
Yes	613	51.9	
Mother’s knowledge of health practices related to pregnancy			
Comprehensive	310	54.5	0.210
Moderate	359	50.4	
Low	220	46.8	
Mother’s knowledge of newborn danger signs			
High	325	43.1	0.001
Moderate	310	53.9	
Low	254	57.5	

### Correlates of early initiation of breastfeeding

As show in Table 3, mode of childbirth was the strongest independent determinant of early initiation of breastfeeding, whereby women who gave birth by caesarean section were 78 % less likely compared to women who gave birth vaginally to initiate breastfeeding within 1 h of birth (adjusted odds ratio (OR) = 0.22, 95 % confidence interval (CI) 0.14, 0.36). Place of childbirth was the other key determinant, with women who gave birth in health facilities being almost twice as likely as women who gave birth at home to initiate breastfeeding within 1 h of birth (OR = 1.75, 95 % CI 1.25, 2.45). Furthermore, ever married women (currently divorced or widowed) were significantly 54 % less likely to initiate breastfeeding within 1 h compared to married women (OR = 0.46, 95 % CI 0.25, 0.87). There was also an issue with district of residence, whereby women in Rufiji district were significantly 48 % less likely than women in Kilombero district to initiate breastfeeding in 1 h of birth (OR = 0.52, 95 % CI 0.31, 0.89). Surprisingly, the lower the mother’s knowledge of newborn danger signs, the higher the odds of early initiation of breastfeeding (moderate knowledge: OR = 1.73, 95 % CI 1.23, 2.42; low knowledge: OR = 2.06, 95 % CI 1.43, 2.96).

**Table 3:** Multivariate logistic regression model of correlates of breastfeeding initiation within 1 h of birth in rural Tanzania in 2011 (*n* = 889)

Covariate	Adjusted Odds Ratio (OR)	*p*-value	95 % Confidence Interval (CI)
Mode of childbirth			
Vaginal (ref)	1.00	−	−
Caesarean section	0.22	<0.001	0.14, 0.36
Place of childbirth			
Home (ref)	1.00	−	−
Health facility	1.75	0.001	1.25, 2.45
Marital status			
Married (ref)	1.00	−	−
Ever married	0.46	0.017	0.25, 0.87
Single	0.83	0.384	0.54, 1.26
District of residence			
Kilombero (ref)	1.00	−	−
Rufiji	0.52	0.016	0.31, 0.89
Ulanga	0.83	0.414	0.52, 1.31
Mother’s knowledge of newborn danger signs			
High (ref)	1.00	−	−
Moderate	1.73	0.001	1.23, 2.42
Low	2.06	<0.001	1.43, 2.96

*Ref* = Reference category or baseline outcome. This model is adjusted for: education attained, pregnancy wantedness, religion, ethnicity, type of residence, whether or not a respondent was counseled on immediate breastfeeding during ANC visits, mother’s knowledge of health practices related to pregnancy, and number of ANC visits made during pregnancy

Women with formal education at secondary or higher levels were almost twice as likely as those without to initiate breastfeeding within 1 h, but the effect was not significant at 5 % (OR = 1.94, 95 % CI 0.89, 4.25). Insignificant also were ethnicity, religion, type of residence, and pregnancy wantedness. Early initiation of breastfeeding was also not affected by knowledge of immediate breastfeeding, number of ANC visits and mothers knowledge of health related practices during pregnancy.

## Discussion

This study assessed prevalence and determinants of early initiation of breastfeeding among 889 women who had recently given birth in Kilombero, Rufiji and Ulanga districts of Tanzania, using cross–sectional household survey data collected in 2011. As highlighted previously, early initiation of breastfeeding is an essential component of maternal and newborn health. Early suckling stimulates the production of breast milk as well as oxytocin which helps the uterus to contract and reduces postpartum blood loss. Colostrum, which is contained in the first breast milk is highly nutritious and has antibodies which protect the newborn baby from acquiring diseases. Moreover, early initiation of breastfeeding enhances bonding between the mother and the child. Therefore early breastfeeding initiation is very crucial for the health of the newborn baby as well as the mother.

Findings from this study reveal that slightly more than one half (51 %) of the women who had given birth within 2 years preceding the survey in the study area initiated breastfeeding within 1 h of birth. This shows presence of a significant proportion of newborn babies in the study area who are not breastfed within 1 h of birth as recommended, and could partly be linked with misconceptions about early breastfeeding as similarly noted in southern Tanzania, that colostrum is dirty and should thus not be given to newborn babies [36]. The observed proportion of early initiation of breastfeeding by this study is comparable to that found in a rural area of Morogoro region in Tanzania [37] as well as the national estimate of 49 % [9], but significantly higher than 18 % reported in southern Tanzania [28]. Early initiation of breastfeeding within Tanzania has been reported to vary between rural and urban areas (45 and 62 % respectively), and ranges from 18 % in Rukwa to 93 % in Manyara region [9]. Outside Tanzania, our observation is comparable to 52 and 53 % observed in Ethiopia [22] and Lesotho [23] respectively, but lower than 58 % observed in Kenya [21] and even lower compared to 95 % recorded in Malawi in 2010 [24].

In the multivariate analysis, childbirth by caesarean section emerged as the strongest factor with negative association with early initiation of breastfeeding. It has been pointed out that postoperative care sometimes takes longer, preventing the mother from achieving contacting her baby during the postpartum period, thus delaying breastfeeding initiation [38]. This association between childbirth by caesarean section and delayed breastfeeding initiation is consistent with a recent systematic review [8] as well as other studies [39–42]. In many low and middle income countries, rates of childbirth by caesarean section are rapidly growing [43]. In Tanzania for example, the current rate of childbirth by caesarean section stands at 5 % [9] and it is 13 % in the current study area from these data. While caesarean section is considered a valuable lifesaving tool for both the mother and newborn in an obstetric emergency [44], interventions that can enhance early initiation of breastfeeding following caesarean section are greatly needed, as well as reducing or if possible stopping caesarean sections that have no medical indication [43]. This is because a caesarean section in one pregnancy suggests the same or at least specialized medical care in subsequent childbirth in order to minimize the risk of complications such as uterus rupture due to the scar from previous caesarean (s). Unfortunately, the fragile health system in the country as the case in many less developed countries may not adequately support this when it is massively needed.

With respect to place of childbirth, women who gave birth in health facilities were more likely than those who gave birth at home to initiate breastfeeding within 1 h of birth. This may be because health workers at health facilities facilitate timely initiation of breastfeeding. This implies that efforts to discourage nonuse of health facilities for childbirth should continue given the detrimental health outcomes associated with the practice, including delayed breastfeeding initiation which consequently increases the risk of newborn morbidity and mortality [8, 26]. Timely initiation of breastfeeding should as well be encouraged for babies born at home. In communities with community health workers (CHWs), which is the case with CHAs in the study area [31], this may be addressed during regular household visits whereby pregnant women can be identified and provided with basic health care and promotion of maternal and newborn health practices.

Maternal knowledge of newborn danger signs was negatively associated with early initiation of breastfeeding, and this observation came up in a dose–response fashion. This was unexpected, as it was anticipated that higher knowledge would increase the likelihood that breastfeeding would be initiated within 1 h of birth. While previous scholars have found that higher educational attainment (not knowledge of newborn danger signs) was associated with higher likelihood of early initiation of breastfeeding [8, 9, 26], the effect of knowledge of newborn danger signs on early initiation of breastfeeding is missing in the previous literature. However, a somewhat similar study in Ghana found that maternal knowledge of at least four newborn danger signs is associated with good neonatal feeding [45]. Ultimately, this study was unable to ascertain the mechanisms fostering this observation, thus warranting further research, especially qualitative studies.

Variations in early initiation of breastfeeding existed between districts, with women in Rufiji district being less likely than those in Kilombero district to initiate breastfeeding early. Women in Ulanga district were similar to those in Kilombero district, possibly because these two districts are in the same geographical location and share a border, thus women therein share the same environment, a situation which may shape their behaviors and health practices alike. Rufiji district, however, is located far from Ulanga and Kilombero, suggesting that variations in environments, cultural values, beliefs, and practices may account for the differences in breastfeeding practice. Although specific explanations of the observed finding are lacking, it has been noted that early initiation of breastfeeding in Tanzania vary by geographical locations [9]. Differences in early initiation of breastfeeding also existed by marital status, with ever married women being less likely to initiate breastfeeding within 1 h than currently married women. Although this was independent of pregnancy wantedness, it remains unclear why this was the case. Further research, especially qualitative studies, are needed to further characterize differential breastfeeding practices and inform interventions to increase early initiation of breastfeeding.

### Limitations

The retrospective nature of this study may have introduced recall bias which may consequently have affected the estimated prevalence of early initiation of breastfeeding in the study area. Also facility level variables such as status of health workers’ training in baby–friendly hospital initiative (BFHI) were not available for inclusion. Furthermore, it is important to understand that cross–sectional designs limit causal inferences since they lack temporal information. The results of this study may not be generalizable nationally or beyond since the data was limited to three districts of Tanzania.

## Conclusions

Despite the known health benefits of early initiation of breastfeeding to the mother and the newborn, only about half of the mothers in Rufiji, Kilombero, and Ulanga districts of Tanzania succeed in starting breastfeeding within 1 h of birth. Efforts should be taken to ensure the universality of this practice. Promotion of health facility use for childbirth should be continued and facilitated to enhance timely initiation of breastfeeding along with promoting other positive health outcomes. On the other hand, timely initiation of breastfeeding should be encouraged for babies born at home. Specifically, developing and implementing interventions to improve timely breastfeeding initiation following childbirth by caesarean section is of utmost importance. Further assessments should occur to understand why higher maternal knowledge of newborn danger signs decreases the odds of early initiation of breastfeeding. Ultimately, early initiation of breastfeeding should be promoted among Tanzanian mothers to ensure healthy outcomes for both mothers and newborns.
